# Expression and Mechanism of TXNIP/NLRP3 Inflammasome in Sciatic Nerve of Type 2 Diabetic Rats

**DOI:** 10.1155/2022/9696303

**Published:** 2022-07-08

**Authors:** Le Han, Guangxia Xi, Na Guo, Jing Guo, Qingfeng Rong

**Affiliations:** Department of Endocrinology for Senior Citizens, Second Hospital of Shanxi Medical University, Taiyuan, 030001 Shanxi, China

## Abstract

**Objective:**

To determine the expression profiling and mechanism of thioredoxin-interacting protein (TXNIP)/nucleotide-binding domain-like receptor protein 3 (NLRP3) inflammasome pathway in sciatic nerve (SN) of type 2 diabetes mellitus (T2DM) rats.

**Methods:**

Ten out of the 35 healthy SD rats (specific pathogen free) purchased were randomized into the control group, while the others were established a T2DM model by feeding a high-fat and high-sugar diet plus laparoscopic injection of 1% streptozotocin (STZ). The successfully modeled rats were subgrouped into two arms: a DM group with 10 rats and a resveratrol- (RES-) treated DM intervention group with 11 rats. Normal saline to control and DM groups. Alterations in fasting blood glucose (FBG) and body weight (BW) at different time points after administration were observed. Sciatic nerve conduction velocity (SNCV) and mechanical pain threshold (MPT) were measured. TXNIP, NLRP3, caspase-1, and interleukin- (IL-) 1*β* levels in rat SN tissue were determined.

**Results:**

DM group rats showed higher FBG and lower BW than control rats at different time points (*P* < 0.05). The FBG of DM intervention group at 2, 4, and 6 weeks after administration was lower, and the BW at 4 and 6 weeks after dosing was higher than DM group. Higher MPT and SNCV were determined in DM intervention group versus DM group (*P* < 0.05). DM group rats had disordered, swollen, and dissolved SN myelin sheath structure; TXNIP inhibition led to a small amount of nerve myelin fragments and mild pathological changes. Lower TXNIP, NLRP3, caspase-1, and IL-1*β* protein levels were found in DM intervention group versus DM group (*P* < 0.05).

**Conclusion:**

The pathogenesis of peripheral neuropathy in T2DM rats may be linked to TXNIP/NLRP3 inflammasome pathway activation, indicating the potential of this pathway as a therapeutic target for diabetic peripheral neuropathy (DPN).

## 1. Introduction

Type 2 diabetes mellitus (T2DM) is the mostly prevalent type of DM in clinic, and its resultant peripheral neuropathy and related complications are important reasons for disability and patient death [1, 2]. Diabetic peripheral neuropathy (DPN) is the result of the interaction of metabolic disorders, vascular damage, abnormal cytokines, oxidative stress (OS), and other factors, in which OS and chronic inflammation play an important part [3]. Therefore, antioxidative inflammatory reaction has become a research hotspot in its treatment. Thioredoxin-interacting protein (TXNIP)/nucleotide-binding domain-like receptor protein 3 (NLRP3), an important axis regulating OS and inflammatory responses, has been proved to interfere with DM, ischemia-reperfusion injury, and chronic inflammation-related diseases [4–6]. Zhou et al. [7] found that TXNIP-mediated NLRP3 inflammasome pathway activation is implicated in nervous system inflammation, and related studies have confirmed the correlation of NLRP3 inflammasome with diabetes and its related complications [7, 8].

Peripheral nerve demyelination and axonal degeneration are the major pathological manifestations of DPN [9]. Previous studies have suggested that diabetic patients may present with terminal nerve symptoms, but not necessarily with distal pathological changes. In recent years, DPN patients have often been associated with proximal nerve involvement. In the study of Jensen et al. [10], hypoglycemia caused by insulin intervention in DPN rats was found to accelerate peripheral neuropathy, and the incidence of SN axonal degeneration was higher, while the possibility of plantar nerve abnormality was lower. However, with the extension of intervention time, the incidence of peripheral degeneration of SN and plantar nerve was equivalent. In addition, Tang et al. [11] observed the morphology of SN in STZ-induced DPN model of rats and found that it was accompanied by demyelination and axonal degeneration. The preceding studies indicate that early DPN is accompanied by both proximal and distal peripheral nerve involvement, while the proximal peripheral nerve involvement shows no obvious symptoms in the early stage, which is not conducive to clinical evaluation and treatment. Currently, the clinical treatment of DPN mainly involves neuronutrition and blood circulation improvement on the basis of blood glucose control, which can only temporarily alleviate the progression of the disease with unsatisfactory results [12]. As medical research advances, molecular targeted therapy has become a research hotspot with remarkable progress achieved in the treatment of malignant tumors, providing a new direction for the research of DPN [10]. Therefore, finding effective intervention targets for DPN is an urgent problem for medical workers.

According to microarray analysis, miR-183 affects neuropathic pain via modulating the TXNIP/NLRP3 inflammasome axis in peripheral nerve injury [13]. However, the expression profiling and mechanism of this axis in the sciatic nerve of type 2 diabetic rats have not been reported. Consequently, the novelty and motivation of this research project is to explore the expression of TXNIP/NLRP3 inflammasome pathway in T2DM rat sciatic nerve (SN) and the related mechanisms, in order to provide reference for targeted therapy of DPN.

## 2. Data and Methods

### 2.1. Laboratory Animal Information

From Beijing Vital River Laboratory Animal Technology (SCXK (Beijing) 2019-0009), 35 healthy male SD rats (specific pathogen free), weighting 200 ± 20 g, were purchased for experiments.

### 2.2. Primary Reagents and Instruments

Reagents and instruments used were streptozotocin (STZ) and resveratrol (RES) (Sigma, USA); Luxol fast blue (LFB; IITC, USA); rabbit anti-TXNIP, NLRP3, caspase-1, and interleukin- (IL-) 1*β* monoclonal antibodies as well as horseradish peroxidase-labeled goat anti-rabbit IgG H&L (CST (China)); blood glucose test paper and tester (Yuwell-Jiangsu Yuyue Medical Equipment & Supply); ND-400 multilead neuromyoelectric evoked potentiometer (Shanghai Poseidon Medical Electronic Instrument); electric Von Frey electronic pain tester (Yishu Technology (Tianjin)); and optical microscope [SZX16, Olympus (China)).

### 2.3. Modeling and Grouping

(1) Modeling: the model was induced by high-fat and high-sugar feeding (basal feed, sucrose, pork, cholesterol, and egg yolk powder with a ratio of 65%, 20%, 10%, 2.5%, and 2.5%, respectively) for 12 weeks, followed by 12 hours of fasting and the subsequent single intraperitoneal injection of 55 mg/kg STZ (0.1 mmol/L citrate buffer). After 72 hours, the rats were fasted for 8 hours. Modeling success was considered if the fasting blood glucose (FBG) was ≥16.7 mmol/L [9]. (2) Grouping: after 7 days of acclimatization, 10 animals were stochastically selected as control group for normal feeding, and the remaining 25 rats were established T2DM model, of which 21 were successfully established with a model success rate of 84%. The modeled rats were randomized into two arms: a DM group with 10 rats and a resveratrol- (RES-) treated DM intervention group with 11 rats. DM intervention group was given 10 mg/kg RES intraperitoneally, once daily, for 6 weeks, while normal saline injection with the same volume was used in the other two groups. The experiment, ratified by the Experimental Animal Ethics Committee of our hospital, complies with the Chinese guidelines for the care and use of laboratory animals.

### 2.4. Fasting Blood Glucose (FBG) and Body Weight (BW) Measurements

FBG was detected on the day of administration, as well as 2, 4, and 6 weeks after administration, and BW was measured. FBG detection method: rats were fasted and deprived of water for 8 hours before detection, and tail tip blood was collected for FBG detection with a glucose meter.

### 2.5. Mechanical Pain Threshold (MPT) and Sciatic Nerve Conduction Velocity (SNCV) Measurements

Two hours after administration, the rats were placed on a raised metal mesh covered with transparent plexiglass. After 15 minutes of adaptation, they were vertically stimulated with an electronic pain meter (with gradually increasing intensity) in the middle of the right rear plantar, and the pressure value (g) was recorded when paw withdrawal reaction occurred. Three repeated measurements were performed for each rat with an interval of >30 s, and the mean value was calculated. The animals were anesthetized by 1% pentobarbital sodium (35 mg/kg) intraperitoneal injection after MPT measurement and fixed supine on the operating table. The temperature in the operating room was controlled at 23 ± 2°C, and the rat body temperature was maintained at about 37°C. Rat SNCV was measured by neuromyoelectric evoked potentiometer: the stimulating needle electrode and the recording needle electrode were inserted into the left ischiatic notch and the SN passing through the ankle joint, respectively, with the reference electrode placed between the two electrodes. The single pulse square wave stimulation mode was adopted, and the parameters were set as follows: wave width: 0.2 ms, intensity: 1.0 times the threshold, and interval between two consecutive stimuli: 5 s. The time from the start of stimulation to the occurrence of evoked potential (T), the distance from the stimulating electrode to the recording electrode (S), and the SNCV (*V* = *S*/*T*) were recorded.

### 2.6. Observation of Pathological Changes of SN by LFB Myelin Staining

After the determination of MPT and SNCV, the rats were anesthetized with pentobarbital sodium (40 mg/kg) and the SN was isolated. The left SN was treated with 10% neutral formalin fixation for 24 hours and paraffin-embedded for morphological examination. The right side was stored in liquid nitrogen at -80°C for Western blot (WB) analysis. Paraffin-embedded samples were then made into 4 *μ*m thick slices and treated with dewaxing, dehydration with gradient alcohol, and 24 hours of staining with 1% LFB. After rinsing with distilled water, 0.05% lithium carbonate solution was added for color separation for 15 s, followed by washing with 70% ethanol and rinsing with distilled water after clear background under optical microscope. The histopathological changes of SN samples were observed microscopically after eosin restaining and neutral gum sealing.

### 2.7. TXNIP, NLRP3, Caspase-1, and IL-1*β* Protein Expressions by WB

The cryopreserved SN tissue was taken out and lysed with cell lysate to collect the total protein, whose content was identified by BCA. The protein was then subjected to SDS-PAGE electrophoresis, membrane transfer, 5% skim milk blocking (1 h), and overnight culture (4°C) with TXNIP, NLRP3, cleaved caspase-1, and IL-1*β* primary antibody diluent (1 : 1000). After that, a horseradish peroxidase-labeled IgG diluted at 1 : 3000 was added for a 1-hour incubation (37°C). ECL chemiluminescence was used for development, fixation, and photography. ImageJ software (National Institutes of Health, Bethesda, MA, USA) analyzed bands' gray values, and the ratio of target band to internal reference protein *β*-actin band gray value was used to represent the protein expression level.

### 2.8. Statistical Processing

The statistical software used in this research was SPSS version 26.0 (SPSS, Chicago, IL, USA), and the variables were recorded as mean ± standard deviation ($\bar{x}\pm s$). Repeated measures data were analyzed by repeated measures ANOVA, the comparison among multiple groups was performed using one-way ANOVA followed by Bonferroni post hoc test, and the comparison between two samples was made by the SNK test, with *P* < 0.05 as the significance level.

## 3. Results

### 3.1. Comparison of FBG and BW

FBG and BW (*F*_intergroup__,_*F*_time_, and *F*_interaction_) showed statistical significance among control, DM, and DM intervention groups (*P* < 0.001); at various time points, FBG was higher and BW was lower in DM group versus control group (*P* < 0.05). DM intervention group showed lower FBG at 2, 4, and 6 weeks after dosing and higher BW at 4 and 6 weeks after administration than DM group (*P* < 0.05). Control and DM groups showed no evident differences in FBG and BW at various time points (*P* > 0.05), while in DM intervention group, FBG decreased and BW increased with time (*P* < 0.05) (Table 1).

### 3.2. Comparison of MPT and SNCV

The comparison of MPT and SNCV revealed statistical significance among control, DM, and DM intervention groups (*P* < 0.001); MPT and SNCV were lower in DM group versus control group (*P* < 0.05). And MPT and SNCV were higher in DM intervention group as compared to DM group (*P* < 0.05) (Table 2).

### 3.3. Observation of LFB Myelin Staining

**Figure 1:**
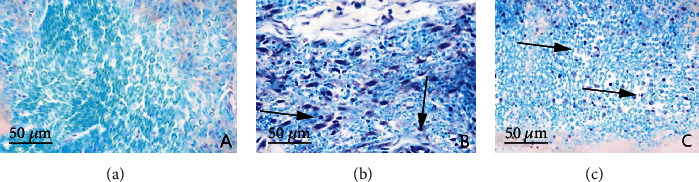
LFB staining results of rat sciatic nerve myelin sheath in control group (a), DM group (b) and DM intervention group (c) (LFB staining, ×400). The arrows indicate diseased parts.

Microscopically, the myelin sheath of SN in control group was blue, the axons were not colored, and the structure was arranged regularly and evenly with a clear outline. In DM group, myelin sheath structure was disordered, and myelin was swollen and dissolved. There was a small amount of nerve myelin debris in DM intervention group, together with myelin swelling and structural disorders that were lighter than those in DM group (Figure 1).

### 3.4. Comparison of Caspase-1, IL-1*β* TXNIP, and NLRP3 Protein Expressions in Rat SN

**Figure 2:**
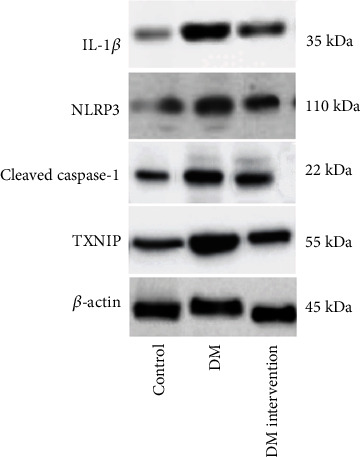
TXNIP, NLRP3, caspase-1, and IL-1*β* protein expressions in rat sciatic nerve by Western blot.

As shown in Table 3 and Figure 2, the three groups showed evident differences in TXNIP, NLRP3, caspase-1, and IL-1*β* protein levels (*P* < 0.001). The protein levels of the above four genes were higher in DM group versus control group (*P* < 0.05) and were lower in DM intervention group compared with DM group (*P* < 0.05).

## 4. Discussion

Hyperglycemia-induced oxidative stress (OS) and chronic inflammation are considered to be the basis of DPN. OS and inflammatory reaction can induce mitochondria to produce reactive oxygen species and activate various metabolic pathways to promote the formation of oxygen free radicals, which results in a vicious circle that activates innate immunity and induces neuroinflammation, triggering DNP [14, 15]. NLRP3 inflammasome, a vital member of the NLRs family, includes caspase-1, NLRP3, and apoptosis-associated speck-like protein containing a CARD (ASC), constituting an important part of the body's innate immunity [16]. NLRP3 is normally inactive in the cytoplasm of cells in homeostasis. When various factors cause changes in the internal environment, NLRP3 recruits ASC and caspase-1 precursors through oligomerization to form multiprotein complexes, induces caspase-1 precursors to self-cleave into activated caspase-1, and cleaves IL-8 and IL-1*β* precursors to produce mature inflammatory cytokines, thus participating in the downstream inflammatory reaction [17, 18]. In addition to directly activating NLRP3 inflammasome, excessive generation of oxygen free radicals can also cause TXNIP and thioredoxin dissociation and promote TXNIP and NLRP3 inflammasome aggregation and activation, which further activates caspase-1 and induces inflammatory cytokines (IL-1*β*, IL-8, etc.), inducing the injury of islet *β* cells and peripheral nerve cells [17, 19].

The results of the T2DM rat model established in this study identified that DM group had higher FBG, lower BW, and decreased MPT and SNCV than control group at different time points after administration. LFB staining showed obvious swelling, dissolution, and structural disorder of SN myelin sheath in DM modeled rats. After the intervention of RES, a TXNIP inhibitor, decreased FBG, increased BW, MPT and SNCV, and alleviated pathological changes of SN myelin sheath were observed in DM model animals. Therefore, antagonizing TXNIP/NLRP3 inflammasome axis could alleviate T2DM-related symptoms and inhibit DNP lesions. Widely expressed in multiple cells, TXNIP is an inhibitor of endogenous active oxygen scavenging protein thioredoxin and an important molecular nutrition sensor for regulating OS and inflammation during energy metabolism [20, 21]. As a known negative modulator of antioxidant protein thioredoxin, TXNIP has emerged as a promising target in a number of diseases like gouty arthritis [22], cervical inflammation [23], and central nervous system-related injuries or diseases [24, 25]. TXNIP is essential in its pathogenesis.

Previous evidence has shown that TXNIP linked OS to NLRP3 inflammasome activation [20], indicating its crucial role in congenital immunity and inflammation. Wang et al. [26] found that TXNIP/NLRP3 inflammasome pathway can mediate angiotensin II-induced apoptosis of islet *β* cells and participate in the occurrence of diabetes. Some scholars [27–30] believe that TXNIP/NLRP3 axis is involved in renal ischemia-reperfusion injury and T2DM cardiomyopathy in diabetic rats. Gu et al. [31] demonstrated that targeting NLRP1 and inactivating reduced nicotinamide adenine dinucleotide phosphate oxidase axis can inhibit cell pyroptosis in diabetic retinopathy. WB measured the protein expression of TXNIP, NLRP3, caspase-1, and IL-1*β* in rat SN. The results showed higher protein levels of the four in DM group versus control group. However, their protein levels decreased in DM intervention group treated with RES. The activation of NLRP3 is influenced by multiple factors or cellular proteins. TXNIP/NLRP3 is the key signal system for NLRP3 inflammasome formation and activation; inhibiting TXNIP markedly decreased NLRP3, caspase-1, and ACS activity as well as IL-1*β* generation [32, 33]. Combining the results of BW, FBG, SN MPT, SNCV, and SN myelin LFB staining in T2DM rats, we can find a close connection between DNP occurrence and TXNIP/NLRP3 inflammasome pathway activation.

This study still has some limitations. Despite the close association between DNP occurrence and TXNIP/NLRP3 inflammasome pathway activation, we demonstrated that the increase of OS may promote cellular inflammation and apoptosis. Thus, more experiments should be conducted to investigate the underlying mechanism of TXNIP/NLRP3 inflammasome pathway on OS.

## 5. Conclusion

TXNIP/NLRP3 inflammasome pathway-associated proteins are abnormally expressed in the SN of T2DM rats, and inhibiting the activation of this pathway can alleviate DNP-related symptoms, so TXNIP/NLRP3 inflammasome pathway can be considered as a therapeutic target for DNP.

## Figures and Tables

**Table 1:** Comparison of rat FBG and body weight among the three groups ($\bar{x}\pm s$).

Groups	Body weight (g)			
	On the day of administration	2 weeks after administration	4 weeks after administration	6 weeks after administration
Control group	455.32 ± 24.35	461.32 ± 28.78	458.63 ± 20.01	459.36 ± 26.74
DM group	360.32 ± 23.32^a^	355.36 ± 26.36^a^	340.12 ± 28.52^a^	340.36 ± 24.30^a^
DM intervention group	345.35 ± 20.63^a^	365.52 ± 22.00^ac^	388.16 ± 24.35^abcd^	412.35 ± 28.35^abcde^
*F* value	*F*_intergroup_ = 15.362, *F*_time_ = 102.332, *F*_interaction_ = 88.451			
*P* value	*P*_intergroup_ < 0.001, *P*_time_ < 0.001, *P*_interaction_ < 0.001			

Groups	FBG (mmol/L)			
	On the day of administration	2 weeks after administration	4 weeks after administration	6 weeks after administration
Control group	4.85 ± 0.68	4.88 ± 0.70	5.01 ± 0.71	4.82 ± 0.65
DM group	22.32 ± 3.23^a^	22.01 ± 3.05^a^	22.85 ± 4.23^a^	24.01 ± 3.43^a^
DM intervention group	22.52 ± 3.40^a^	16.64 ± 3.03^abc^	10.33 ± 2.01^abcd^	8.62 ± 1.22^abcde^
*F* value	*F*_intergroup_ = 21.320, *F*_time_ = 92.312, *F*_interaction_ = 64.350			
*P* value	*P*_intergroup_ < 0.001, *P*_time_ < 0.001, *P*_interaction_ < 0.001			

Note: ^a,b^*P* < 0.05 compared with the control group and the DM group, respectively; ^c^*P* < 0.05 vs. on the day of administration; ^d^*P* < 0.05 vs. 2 weeks after administration; ^e^*P* < 0.05 vs. 4 weeks after administration.

**Table 2:** Comparison of mechanical pain threshold and sciatic nerve conduction velocity of rats among the three groups ($\bar{x}\pm s$).

Groups	Mechanical pain threshold (g)	Sciatic nerve conduction velocity (m/s)
Control group	75.32 ± 7.52	55.32 ± 6.23
DM group	40.12 ± 5.32^a^	40.12 ± 5.77^a^
DM intervention group	60.21 ± 6.80^ab^	48.78 ± 5.02^ab^
*F* value	71.243	18.075
*P* value	<0.001	<0.001

Note: ^a^*P* < 0.05 vs. control group; ^b^*P* < 0.05 vs. DM group.

**Table 3:** Comparison of protein expression of TXNIP, NLRP3, caspase-1, and IL-1*β* in rat sciatic nerve among the three groups ($\bar{x}\pm s$).

Groups	TXNIP	NLRP3	Caspase-1	IL-1*β*
Control group	0.50 ± 0.10	0.33 ± 0.05	0.20 ± 0.04	0.10 ± 0.03
DM group	1.35 ± 0.20^a^	0.75 ± 0.08^a^	0.40 ± 0.05^a^	0.92 ± 0.10^a^
DM intervention group	0.71 ± 0.12^ab^	0.48 ± 0.06^ab^	0.32 ± 0.04^ab^	0.22 ± 0.05^ab^
*F* value	92.875	109.437	53.681	450.233
*P* value	< 0.001	< 0.001	< 0.001	< 0.001

Note: ^a,b^*P* < 0.05 compared with the control group and the DM group, respectively.

## Data Availability

The labeled datasets used to support the findings of this study are available from the corresponding author upon request.
